# Breaking the Chains: Systemic Barriers to Human Rights-Based Mental Health Services

**DOI:** 10.1192/j.eurpsy.2026.10978

**Published:** 2026-07-31

**Authors:** J. K. Stefansson, S. Olafsdottir

**Affiliations:** Faculty of Sociology, Anthropology and Folkloristics, University of Iceland, Reykjavik, Iceland

## Abstract

**Introduction:**

Autonomy, participation, and empowerment are central to international human rights frameworks, especially the UN Convention on the Rights of Persons with Disabilities. Yet a persistent gap remains between these ideals and users’ lived experiences, often marked by coercion, conditional supports, and tokenistic involvement. This study explores whether such discrepancies arise not only from implementation gaps but from institutional logics and power relations within mental health care. Using Institutional Logics Theory and Relational Constructivism, we analyse how organisational routines shape autonomy and empowerment, and why rights-based reforms often remain rhetorical.

**Objectives:**

Despite policy efforts to align mental health services with human rights frameworks, principles such as autonomy, reciprocity, and empowerment remain far from realised in practice. This study explores how institutional logics and power dynamics shape service users’ experiences in Iceland, with a focus on barriers to rights-based, recovery-oriented care.

**Methods:**

Thirteen semi-structured interviews were conducted with long-term service users in Iceland. Participants were purposively sampled to capture diverse experiences across service settings. Data were analysed using reflexive thematic analysis, guided by Relational Constructivism and Institutional Logics Theory, which enabled examination of how macro-level institutional logics and micro-level power dynamics intersect in everyday encounters.

**Results:**

The biomedical model dominated services, reframing lived experience as pathology, positioning medication as the default, and embedding rigid hierarchies. This curtailed autonomy, sidelined trauma and social context, and undermined trust in users’ knowledge. Analysed through Relational Constructivism, accounts showed two forms of power: *instructive power* steered choices through selective information, persuasion, and tokenistic consultation; *destructive power* reduced possibilities by tying housing, welfare, or family contact to compliance, or by enforcing treatment against users’ will. Together these dynamics foreclosed autonomy as a relational achievement and rendered empowerment largely rhetorical. Still, participants reported positive encounters with staff, trainees, and peer-support organisations, where respect and equality created genuine spaces for participation, though such practices remain marginal.

**Conclusions:**

Reform requires more than policy commitments or symbolic participation. As long as decision-making authority stays concentrated in professional and bureaucratic hands, empowerment will remain rhetorical. Change is needed to grant users formal power in decisions, decouple welfare from psychiatric compliance, and embed peer- and relation-based approaches in service delivery. Without such transformation, human rights in mental health care risk remaining celebrated ideals but unrealised in practice.

**Disclosure of Interest:**

None Declared

